# The “*Artificial Artery*” as *In Vitro* Perfusion Model

**DOI:** 10.1371/journal.pone.0057227

**Published:** 2013-03-07

**Authors:** Doreen Janke, Joachim Jankowski, Marieke Rüth, Ivo Buschmann, Horst-Dieter Lemke, Dorit Jacobi, Petra Knaus, Ernst Spindler, Walter Zidek, Kerstin Lehmann, Vera Jankowski

**Affiliations:** 1 Charité-Universitaetsmedizin Berlin, Julius Wolff Institute and Berlin-Brandenburg Center for Regenerative Therapies (CVK), Berlin, Germany; 2 Charité-Universitaetsmedizin Berlin, Medizinische Klinik IV (CBF), Berlin, Germany; 3 eXcorlab GmbH, Industrie Center Obernburg, Obernburg, Germany; 4 Charité-Universitaetsmedizin Berlin, Center for Cardiovascular Research (CCM), Berlin, Germany; 5 Institute for Chemistry and Biochemistry, FU Berlin, Berlin, Germany; Centro Cardiologico Monzino, Italy

## Abstract

Metabolic stimuli, pressure, and fluid shear stress (FSS) are major mediators of vascular plasticity. The exposure of the vessel wall to increased laminar FSS is the main trigger of arteriogenesis, the remodelling of pre-existent arterio-arteriolar anastomoses to functional conductance arteries. In this study, we have used an in vitro bioreactor to investigate cell-specific interactions, molecular mechanisms as well as time-dependent effects under laminar FSS conditions. This bioreactor termed “artificial artery” can be used for screening potential arterio-protective substances, pro-arteriogenic factors, and for investigating biomarkers of cardiovascular diseases such as cardiac diseases. The bioreactor is built up out of 14 hollow fiber membranes colonized with endothelial cells (HUVECs) on the inside and smooth muscle cells (HUASMCs) on the outside. By means of Hoechst 33342 staining as well as immunocytochemistry of ß-catenin and α-smooth-muscle-actin, a microporous polypropylene membrane was characterized as being the appropriate polymer for co-colonization. Defined arterial flow conditions (0.1 N/m2 and 3 N/m2), metabolic exchange, and cross-talk of HUVECs and HUASMCs through hollow fibers mimic physiological in vivo conditions of the vasculature. Analysing mono- and co-culture secretomes by MALDI-TOF-TOF mass spectrometry, we could show that HUVECs secreted Up4A upon 3 N/m2. A constant cellular secretion of randomly chosen peptides verified viability of the “artificial artery” for a cultivation period up to five days. qRT-PCR analyses revealed an up-regulation of KLF2 and TIMP1 as mechano-regulated genes and demonstrated arterio-protective, homeostatic FSS conditions by a down-regulation of EDN1. Expression analyses of VWF and EDN1 furthermore confirmed that RNA of both cell types could separately be isolated without cross-contamination. CCND1 mRNA expression in HUVECs did not change upon FSS indicating a quiescent endothelial phenotype. Taken together, the “artificial artery” provides a solid in vitro model to test pharmacological active compounds for their impact on arterio-damaging or arterio-protective properties on vascular response.

## Introduction

Cardiovascular diseases (CVDs) are the leading cause of diseases and death in the world. In 2008, it was estimated that 17.8 million people died from CVDs, mainly coronary heart diseases and stroke [1]. In 2030, approximately 23.6 million patients will be suffering from CVDs. The majority of CVDs is avertable through physical activity, a healthy diet, and avoiding tobacco. Nonetheless, preventive measures by use of biomarkers are still insufficient [2], [3]. Therefore, there is a strong need for the identification of underlying yet unknown biomarkers and mediators involved in CVDs for both the therapy of early stage CVDs and the establishment of new therapies in the future. To identify underlying pathways and key players involved, *in vitro* models for both the generation of sufficient amounts of biomarkers and mediators and the testing of new therapeutic agents before animal-based studies are essential. Biomarkers and mediators involved in CVDs have to be fractionated by chromatographic methods before identification. Since the recovery rate of chromatographic methods is strongly limited, the use of *in vitro* models should be appropriate to generate larger amounts of biomarkers to ensure their identification e.g. by mass spectrometry. Many mediators do not cause acute but long-term effects necessitating *in vitro* models with long-term viability and biomechanical response mimicking the *in vivo* situation.

Cell based bioreactors may be an appropriate approach for the generation of biomarkers and mediators involved in the genesis and progression of CVDs. Underlining the importance of the topic, several bioreactors have already been described in the literature. Takei *et al.* [4] e.g. have developed an *in vitro* bioreactor to investigate spontaneous tube formation. Bovine carotid artery vascular endothelial cells (BECs) are colonized into a tube-shaped hollow space surrounded by type I collagen gel. Initiated by VEGF (vascular endothelial growth factor) stimulation, a capillary-like network was formed spontaneously by BECs migrating into the collagen gel. This approach is a good starting point to create capillary-like networks *in vitro* and could be the basis for the potential construction of 3D organs, but it lacks of two aspects comparing it to the *in vivo* situation: Takei *et al.* have used endothelial cell mono-cultures instead of co-culture systems also including stromal cells. Nevertheless, heterotypic cell-cell interactions are critical for the stabilization and proper functioning of native vessels [5], [6]. Creating a curved vascular like structure would furthermore have enabled the volume occupied by the tube-shaped hollow space to be increased [7]. Bishop *et al.* therefore developed an *in vitro* co-culture system consisting of endothelial cells and fibroblasts which allows for a scaffold to build capillary-like networks via angiogenesis [8]. Comparing the morphology of tubules formed using this approach to those of matrigel assays revealed a higher analogy to tubules formed in a microvascular bed *in vivo* [9]. However, cell-cell and cell-matrix interactions are critical and still limited in many of the *in vitro* models used. Although controlling specific interactions in simplified assays is feasible, two-dimensional controlled *in vitro* models of the *in vivo* environment have been difficult to realize [10]. To find a bridge to the *in vivo* environment, several organ culture assays such as the rat aortic ring assay [11] have been developed. The rat aortic model offers the benefit of culturing endothelial cells in the context of native stromal cells and matrix to more closely mimic the *in vivo* environment. Nonetheless, this assay is restricted by a limited viability of the cells and not well defined laminar shear stress conditions highlighting the need for co-culture systems which enable the application of pre-defined flow conditions [12]. Tissue engineered vascular like constructs established under defined FSS conditions turned out as promising approach for patients not having autologous vessels suitable to be used as small diameter grafts [13], [14]. Even though many new approaches showed great promise [15], [16], [17], [18], [19], neither of them could fulfil all requirements for mechanical strength and stiffness to functionally replace a native vessel *in vivo*. 3-dimensional bioreactor systems have been developed to further understand the process of tissue growth equivalent to the *in vivo* situation. Those systems provide the possibility to apply defined mechanical conditions and to determine the stiffness of the developed constructs. They furthermore facilitate long-term culture and scale-up by defined nutrient and oxygen conditions [20], [21], [22]. Nowadays, there are simple devices mimicking one aspect of the cardiovascular environment [23], [24], [25] or more elaborate systems that seek to mimic the vascular environment [26], [27], [28], [29]. However, existing *in vitro* approaches failed to screen potential arterio-protective substances, pro-arteriogenic factors, and to investigate biomarkers of CVDs. They additionally lack the generation of sufficient amounts of biomarkers and mediators which could be analysed by mass spectrometry for instance.

Therefore, we have developed an *in vitro* bioreactor termed “*artificial artery*”. This bioreactor is a co-culture system consisting of endothelial cells (HUVECs) and vascular smooth muscle cells (HUASMCs) to mimic the *in vivo* situation of the vasculature. On the basis of cell proliferation assays and immunocytochemistry analyses, a co-culture medium was defined to feed both cell types with sufficient nutrients and oxygenation. To be in the range comparable to the diameter of smaller arteries *in vivo* [30], [31], we have chosen hollow fiber membranes with a diameter of 330 µm. A multi channel peristaltic pump was used to generate constant low laminar (0.1 N/m^2^) and high laminar FSS (3 N/m^2^) conditions within the biologically relevant range of steady-state fluid mechanical wall shear stress [32], [33]. Based on the conditions of the microporous hollow fiber membrane, cross-talk and metabolic exchange between both cell types comparable to the *in vivo* situation of the vasculature is achieved within a bioreactor module. In this study, we used the “*artificial artery*” to investigate cell-specific interactions, molecular mechanisms as well as time dependent effects under defined shear stress conditions.

## Materials and Methods

### Chemicals

HPLC water (gradient grade) and acetonitrile were purchased from Merck; all other substances are from Sigma Aldrich unless otherwise indicated.

### Cultivation of endothelial cells and vascular smooth muscle cells

Cryopreserved human umbilical vein endothelial cells (HUVECs) and cryopreserved human umbilical artery smooth muscle cells (HUASMCs) were purchased from PromoCell (C-12203, C-22050). Both primary cell types were cultured in “SMC growth medium 2” (PromoCell) supplemented with 0.05 ml/ml FCS, 0.5 ng/ml recombinant human EGF, 2 ng/ml recombinant human bFGF, 5 µg/ml recombinant human insulin, 100 U/ml penicillin, and 100 µg/ml streptomycin (StemCell Technologies). 0.4% (v/v) endothelial cell growth supplement plus heparin (ECGS/H, PromoCell) was additionally supplemented to induce a growth-promoting effect on endothelial cells. Cells were cultured in 37°C, humidified 5% CO_2_ incubators. Both cell types were used at passage 2 to 5.

### Cell proliferation assay

HUVECs and HUASMCs were seeded into 6-well plates at a cell density of 100,000 cells per well. To define optimal conditions for culturing both cell types simultaneously, HUVECs and HUASMCs were cultured in “endothelial cell growth medium”, “SMC growth medium 2” and “SMC growth medium 2” plus ECGS/H. HUVECs and HUASMCs cultured in each medium condition were trypsinized and counted to investigate proliferation after one day and five days of cultivation, respectively.

### Technical aspects of the bioreactor

**Figure 1:**
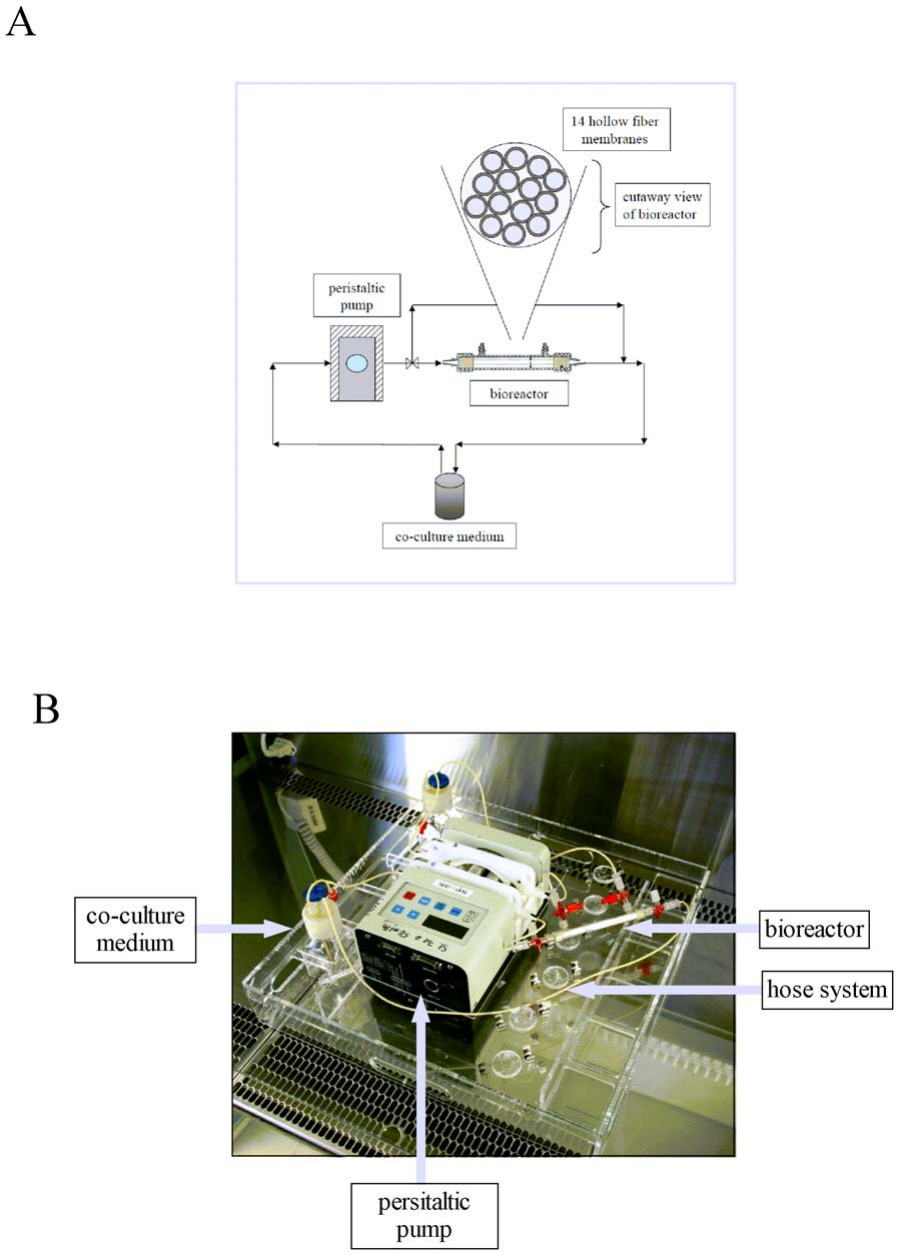
Illustration of the “*artificial artery*” as *in vitro* co-culture model. (**A**) Scheme of the bioreactor “*artificial artery*” consisting of a bundle of polypropylene hollow fiber membranes in a miniaturized and parallelized format. (**B**) Photography of the whole “*artificial artery*” system with a flask of co-culture medium, a peristaltic pump station to apply defined shear stress, and the bioreactor itself.

A module of the “*artificial artery*” is build up out of 14 parallel-arranged hollow fibers of hydrophobic polypropylene membranes (**Figure 1A**). Luminal and ablumial ports enable feeding of both cell types with eutrophic co-culture medium (**Figure 1A**). An affiliated peristaltic pump station ensures that pre-defined FSS conditions can be applied to the lumen of hollow fibers colonized with endothelial cells mimicking the *in vivo* situation of the vessel wall (**Fig. 1B**). Hollow fiber modules were provided by Membrana. Untreated modules consist of 14 Plasmaphan P1LX fibers (polypropylene) with an effective inner diameter of 330 µm and a wall thickness of 150 µm. A transmembrane flow (isopropylalcohol, 37°C) of 9.3 ml/[min×cm^2^×bar] and a maximum pore size of 0.47 µm provide all important membrane performance characteristics. The active membrane area inside is 0.07 mm^2^ and the active membrane area outside is 1.4 mm^2^. The active membrane length is 50 mm and the filling degree of hollow fiber modules is 34.7%. The hollow fiber bundles were fixed within the bioreactor housing (polycarbonate) using a highly viscous polyurethane solution as potting material. Following its penetration between hollow fibers and housing material, the polyurethane solution polymerized. After separating the inner and outer fiber compartments, an exchange of fluids was only realizable through hollow fiber membrane pores. Each module was sterilized by wet steam (121°C for 15 min).

### Hollow fiber settlement of mono-culture modules

Untreated hollow fiber modules were rinsed with 70% ethanol. After washing with double distilled water, the modules were equilibrated with phosphate-buffered saline (PBS, with calcium/magnesium) for 48 h at 37°C under low laminar flow conditions (0.1 N/m^2^). Before seeding HUVECs within mono-culture modules, the lumen of the hollow fiber membranes was coated with fibronectin (10 µg/ml, diluted in PBS) (Sigma-Aldrich) for 1.5 h at 37°C under static conditions. After equilibrating the modules with “SMC growth medium 2” plus ECGS/H for 1 h under static conditions, HUVECs were seeded at 2×10^5^ cells/cm^2^ in a volume of 2.5 ml at the inner cellular space of hollow fiber modules. Following attachment for 1 h under static conditions, the cells were supplied with “SMC growth medium 2” plus ECGS/H at low laminar abluminal flow (0.1 N/m^2^) for 2.5 h at 37°C. Shear stress was then applied at 0.1 N/m^2^ (control) or 3 N/m^2^ for five days, respectively. To improve attachment within HUASMC mono-cultures, the outer side of hollow fibers was coated with fibronectin and incubated with “SMC growth medium 2” plus ECGS/H as described above. HUASMCs (2×10^5^ cells/cm^2^ in a volume of 2.5 ml) were inoculated at the outer cellular space of hollow fiber modules. The cells were allowed to attach for 1.5 h at 37°C under static conditions before applying laminar FSS over a five day period. Confluence of both cell types was achieved after one day. An IPC multi channel peristaltic pump (IDEX Ismatec GmbH, Germany) was used to generate constant low laminar (0.1 N/m^2^) and high laminar FSS (3 N/m^2^) conditions. Within a period of five days, 20 ml “SMC growth medium 2” plus ECGS/H was added freshly each day to provide both cell types with sufficient nutrients and oxygenation. The secretome of each module was also isolated daily before continuing FSS stimulation.

Laminar FSS was calculated as follows:
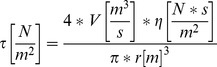
here τ is the FSS, V is the flow rate within a single hollow fiber, r is the radius of a hollow fiber and η is the medium viscosity.

### Hollow fiber settlement of co-culture modules

To reach simultaneous confluence of HUVECs and HUASMCs within hollow fiber modules, the hollow fibers were equilibrated as described for mono-culture systems. The lumen of hollow fibers was coated with fibronectin and incubated with “SMC growth medium 2” plus ECGS/H comparable to mono-culture conditions. HUVECs were seeded at the inner cellular space of hollow fiber modules. After 1 h, the cells were supplied with “SMC growth medium 2” plus ECGS/H at low laminar abluminal flow (0.5 N/m^2^) for 2.5 h at 37°C. Following abluminal fibronectin coating, HUASMCs were inoculated at the outer cellular space of hollow fiber modules for co-colonization experiments. The cells were allowed to attach for 1.5 h at 37°C under static conditions. Shear stress was then applied at 0.1 N/m^2^ (control) or 3 N/m^2^ for five days, respectively.

### Hoechst staining of settled hollow fibers

Hollow fibers were rinsed with PBS and incubated with 2.5% glutaraldehyde (Merck) for 3 min at room temperature to fix human primary cells. After washing the fibers with PBS, cells were stained with 10 µg/ml Hoechst 33342 (Molecular Probes) diluted in PBS for 5 min at room temperature. After washing the fibers twice, they were cut with a razor blade to investigate the human primary cell seeding at the inner and outer space of hollow fibers.

### Scanning electron microscopy

HUVECs grown in polypropylene hollow fibers were fixed with 2.5% glutaraldehyde in PBS, postfixed with 1% osmium tetroxide in PBS for 1 h on ice, dehydrated in a graded series of ethanol, and critical-point-dried from CO_2_. Finally, the samples were sputter-coated with a layer of 7 nm gold/palladium (Bal-Tec) and examined at 20 kV accelerating voltage in a Hitachi S-800 field emission scanning electron microscope.

### Immunocytochemistry of human primary cells and confocal microscopy

Immunocytochemistry was carried out as described previously [34]. Briefly, cells grown in hollow fibers were fixed with 4% paraformaldehyde (Merck) and permeabilized in 0.2% Triton X100/PBS for 5 min at room temperature. Blocking was carried out in 0.2% cold fish gelatine/PBS for 20 min at room temperature. A purified mouse anti-Cadherin-5 antibody (250 µg/ml) (BD transduction laboratories) diluted 1∶200 in 0.2% gelatine/PBS was applied for 1 h at room temperature as marker for HUVECs. After washing the fibers three times with 0.2% gelatine/PBS, an Alexa fluor 488-conjugated donkey anti-mouse antibody (2 mg/ml) (Invitrogen) diluted 1∶200 was applied for 20 min at room temperature. HUASMCs were stained with a Cy3-conjugated mouse anti-α-smooth-muscle-actin antibody (Sigma) diluted 1∶200 for 1 h at room temperature. Cell nuclei were counterstained with Hoechst 33342 as described above. The fibers were washed twice, rinsed with ddH_2_O, and cut open with a razor blade. After mounting the fibers on slides using Fluoromount-G (Southern Biotech), pictures were taken using the upright confocal microscope Leica DM 2500 and a 40x oil immersion objective.

### Isolation of dinucleoside polyphosphates

The (patho-) physiological relevance of the model was validated by quantification of Uridine adenosine tetraphosphate (Up_4_A) in the secretomes of mono- and co-cultures upon FSS stimulation. Up_4_A has been identified as a potent endothelium-derived contracting factor [35] which is involved in the modulation of vascular function by actions on endothelial and smooth muscle cells [36]. *In vivo*, HUVEC cells were capable of synthesizing Up_4_A after FSS stimulation. It could also been shown that Up_4_A is bound by surface receptors of HUVECs to initiate endothelial cell proliferation [35], [37]. For deproteination of mono- and co-culture secretomes, 65 µl perchloric-acid (70%) (Merck) per ml cell-supernatant was added at 4°C. The supernatant was centrifuged at 1500×*g* for 10 min at 4°C. The pH was adjusted to 12 using 5 M KOH (Merck). Mono- and co-culture supernatants were initially fractionated by preparative reversed phase chromatography. P(1),P(5)-diguanosine pentaphosphate (2 µg) was added to the supernatant as an internal standard. Triethylammonium acetate (TEAA, 40 mmol/l) was also added and the pH was titrated to 6.5 with HCl. To concentrate the supernatant of co-cultured cells as well as mono-cultures, it was loaded on a C18 preparative reversed-phase column (Chromolith® Performance RP-18 100-4.6) (Merck). The binding molecules were eluted with 30% acetonitrile (ACN) in water at a flow rate of 1.0 ml/min. The elution was detected by measuring the UV absorption at 254 nm.

The eluate of the preparative reversed-phase chromatography column was further purified with an affinity chromatography as earlier described [35]. Briefly, the pH of the eluate from the preparative reversed-phase chromatography was adjusted to pH 9.5 with ammonium acetate and loaded to the affinity column. The column was washed with an ammonium acetate solution with a flow rate of 3.0 ml/min. Binding substances were eluted with 10 mmol/l HCl solution. The elution was monitored by measuring the UV absorption at 254 nm. To desalt the eluate of the affinity chromatography, it was injected into a reversed phase high performance liquid column (Chromolith RP-18e 100-4.6) (Merck). After removing substances not binding to the column with aqueous 40 mmol/l TEAA, the absorbed substances were eluted with 30% ACN in water at a flow rate of 1.0 ml/min. The elution was monitored by measuring UV absorption at 254 nm.

### Matrix assisted laser desorption/ionisation mass spectrometry

The lyophilised fractions of the reverse-phase chromatography were analysed by matrix-assisted laser desorption/ionisation mass spectrometry (MALDI-MS) [38], [39], [40]. 1 µl of each fraction was prepared on a pre-structured MALDI sample support (MTP AnchorChip™ 400/384) (Bruker-Daltonics) [41] and dried gently on an inert metal surface before introduction into the mass spectrometer. Mass spectrometric measurements were performed on a Bruker Ultraflex-III TOF/TOF instrument (Bruker-Daltonic). Mass spectra of positively charged ions were analysed in the reflector mode using delayed ion extraction. Fragment ion spectra were recorded using the LIFT option of the instrument. The dinucleoside polyphosphate diguanosine pentaphosphate (Gp_5_G) was added to the sample as internal standard in the case of kinetic measurements by using MALDI-MS.

### Secretome analysis

Mono- and co-culture secretomes were fractionated by preparative reversed phase chromatography as described above. The lyophilised samples were concentrated and desalted utilizing the PerfectPure C-^18^ ZipTip™ (Millipore Corporation) technology using water with 0.1% trifluoroacetic acid (TFA), and directly eluted with 60% acetonitrile in water with 0.1% TFA. The eluate was spread onto the MALDI target plate (MTP ground steel 400/384) (Bruker-Daltonic) using a-cyano-4-hydroxycinnamic acid (2.5 mg/ml) as matrix. MALDI-MS was performed as described above.

### RNA isolation and quantification

Trizol (Invitrogen) was applied luminally or abluminally of hollow fibers. RNA was isolated according to the manufacture's protocol. RNA quantity and purity was measured using the NanoDrop ND-1000 spectrophotometer (Thermo-Fisher). RNA quality was analysed via agarose gel electrophoresis.

### Semi-quantitative real-time reverse transcription-PCR

cDNA synthesis was performed using 1 µg RNA as described in the manual of the “High capacity cDNA Reverse Transcrition kit with RNase Inhibitor” (Applied Biosystems). Semi-quantitative real-time reverse transcription-PCR (qRT-PCR) was used to analyse differential expression of FSS induced genes (KLF2 and TIMP1), genes specifically expressed in endothelial cells (VWF and EDN1), and CyclinD1, a marker for proliferation. Briefly, amplification of cDNA was carried out in a iQ™5 real-time PCR detection system (BioRad) using Power SYBR green (Applied Biosystems). The PCR profile was 95°C for 10 min; 40 cycles of 95°C for 15 s, 60°C for 15 s, and 72°C for 30 s; 95°C for 1 min, 55°C for 1 min; 81 cycles of 55°C for 10 s, and continuously 10°C. qRT-PCR was performed using the following custom primers (Invitrogen): RPLPO: forward primer: 5′-ACGGGTACAAACGAGTCCTG-3′, reverse primer: 5′-AGCCACAAAGGCAGATGGAT-3′; KLF2: forward primer: 5′-CTTTCGCCAGCCCGTGCCGCG-3′, reverse primer: 5′-AAGTCCAGCACGCTGTTGAGG-3′; TIMP1: forward primer: 5′-AATTCCGACCTC GTCATCAG-3′, reverse primer: 5′-GTTGTGGGACCTGTGGAAGT-3′; CCND1: forward primer: 5′-CCCTCGGTGTCCTACTTCAA-3′, reverse primer: 5′-CTCCTCGCACTTCTGTTCCT-3′; EDN1: forward primer: 5′-CCAAGGAGCTCCAGAAACAG-3′, reverse primer: 5′-GATGTCCAGGTGGCAGAAGT-3′; VWF: forward primer: 5′-TGGGAGCATGTACAGCTTTG-3′, reverse primer: 5′-GGCTCACTCTCTTGCCATTC-3′. Results were quantified using the ΔΔ Ct method. Gene expression data were normalised against the housekeeping gene acidic ribosomal phosphoprotein PO (RPLPO).

### Statistical methods

Data are given as mean values derived from three independent biological replicates with standard error mean. All statistical analyses were done using SPSS software (Microsoft SPSS for Windows, version 12.0). The Wilcoxon-Mann-Whitney test was used for non-parametric statistical tests. p<0.05 (two-sided) was considered to indicate statistical significance.

## Results

Our *in vitro* co-culture system is a model to investigate and/or generate mediators and biomarkers for improved prediction, improved treatment, and optimized therapeutic strategies of CVDs such as diabetes mellitus and endothelial dysfunction. We therefore named the model “*artificial artery*”. In this model, human primary umbilical vein endothelial cells (HUVECs) were colonized on the inner side of hollow fiber membranes and human primary umbilical artery smooth muscle cells (HUASMCs) on the outer side of the hollow fibers.

To define optimal growth conditions for HUVECs and HUASMCs within a hollow fiber module, we have investigated their proliferation and expression of cell specific markers under different medium conditions such as “endothelial cell growth medium” (EGM), “SMC growth medium 2”, and “SMC growth medium 2” plus ECGS/H. Based on cell proliferation assays, we found “SMC growth medium 2” plus ECGS/H as the optimal medium for culturing both cell types simultaneously. These data were supported by analysing the expression of platelet endothelial cell adhesion molecule (PECAM1) as endothelial cell specific marker and α-smooth-muscle-actin as marker for smooth muscle cells via immunocytochemistry.

In order to find the optimal membrane type for co-colonization experiments, we have tested different flat sheet membranes made of polyamide, polyethersulfone, polyethylene, polyetherester, polyethylene terephthalate, polytetrafluoroethylene, and polypropylene. We have chosen those materials because they are known to be used clinically and in research [42], [43], [44], [45], [46], [47]. All membranes were provided by Membrana and varied in their physical properties such as wall thickness and maximum pore size (**Table S1**). To validate confluent cell seeding onto polymer flat sheet membranes, HUVEC and HUASMC nuclei were stained with Hoechst 33342. Polyethersulfone and polypropylene were found to be appropriate for the colonization with human primary cells (**Figure S1A**). Membrana furthermore provided hollow fibers made of the same materials with an effective inner diameter of 300 and 330, respectively. In order to find an appropriate cell number, varying cell seeding concentrations were tested, whereby 2×10^5^ cells/cm^2^ was found to be the optimal concentration for co-colonization experiments (data not shown). To further optimize the attachment of human primary cells onto the membrane surface, membranes were coated with gelatin, collagen or fibronectin, respectively. Cell nuclei staining revealed an optimal cell attachment on membranes coated with 10 µg/ml fibronectin. To exclude negative side-effects of the fibronectin coating on human primary endothelial cells, the secretome of these cells grown onto coated and uncoated membranes was analysed by MALDI-TOF/TOF mass spectrometry. Only marginal differences were ascertained between the secretome of cells grown onto uncoated or fibronectin-coated membranes (**Figure S1B** **and S1C**). To investigate if human primary cells maintain their phenotype onto pre-selected polyethersulfone or polypropylene membranes, specific immonocytochemistry staining of markers for both cell types were performed following different membrane washing procedures. β-catenin is a key downstream effector in the Wnt signaling pathway [48] and was used to demonstrate cytoskeletal alignment, cell attachment, and cell-cell contacts in endothelial cells (**Figure S1D**). α-smooth-muscle-actin is known as contractile protein specifically expressed in smooth muscle cells [49]. Taken together, a microporous polypropylene membrane coated with fibronectin was characterized as being the appropriate polymer for co-colonization of HUVECs and HUASMCs.

**Figure 2:**
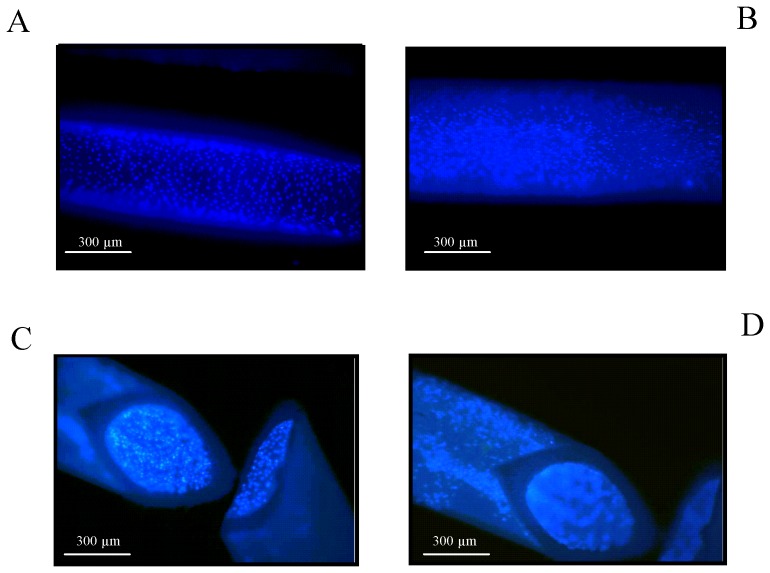
Hoechst 33342 staining of human primary cell nuclei colonized onto polypropylene hollow fiber membranes. (**A**) Longitudinal section of the inside of a hollow fiber membrane confluently colonized with HUVECs and stained with Hoechst 33342 after 0.1 N/m^2^ applied for 24 h (magnification: 1∶100). (**B**) Longitudinal section of the outside of a HUASMC mono-culture module stimulated with low laminar FSS for 24 h (magnification: 1∶100). (**C**) Cross-section of a polypropylene hollow fiber co-colonized with HUVECs and HUASMCs. In focus are HUVECs on the inside upon 0.1 N/m^2^ applied for five days (magnification: 1∶100). (**D**) Cross-section of a co-culture module showing HUASMCs on the outside of a hollow fiber after low laminar FSS stimulation for five days (magnification: 1∶100).

For validating the model, Hoechst 33342 was used to stain DNA of HUVECs and HUASMCs. Longitudinal sections of mono-culture hollow fiber modules stimulated with 0.1 N/m^2^ for 24 h demonstrate a confluent colonization of HUVECs on the inner side (**Figure 2A**) and HUASMCs on the outer side (**Figure 2B**). Cross-sections of co-culture modules simultaneously colonized with both cell types reveal the parallel confluence of HUVECs (**Figure 2C**) and HUASMCs (**Figure 2D**) after a cultivation period of five days.

**Figure 3:**
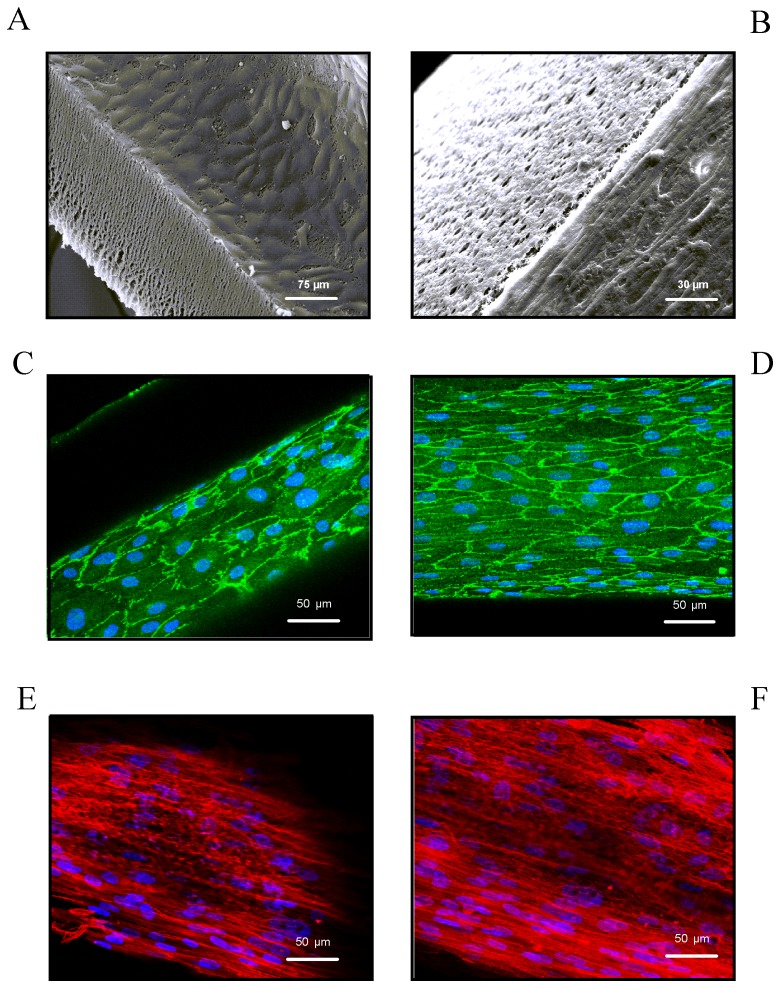
Morphological pictures of HUVEC/HUASMC co-cultures upon physiological FSS conditions. (**A**) Scanning electron microscopy picture of the homogenously colonized inside of a hollow fiber with confluently grown and characteristically cobblestone shaped human primary endothelial cells upon the application of low laminar FSS (0.1 N/m^2^) for 24 h (magnification: 1∶500). (**B**) Scanning electron microscopy picture of HUASMCs on the hollow fiber outside with their typical cell cytoskeletal structure and morphology upon 0.1 N/m^2^ applied for 24 h (magnification: 1∶400). (**C**) Confocal microscopic immunolocalization of Cadherin-5 in co-cultivated HUVECs exposed to low laminar FSS (0.1 N/m^2^) over a period of five days (magnification: 1∶400). (**D**)Cadherin-5 in HUVECs upon high laminar FSS (3 N/m^2^) (magnification: 1∶400). (**E**) Confocal microscopic immunolocalization of α-smooth-muscle-actin in co-cultivated HUASMCs upon 3 N/m^2^ luminally applied for a five day period (magnification: 1∶400). (**F**) α-smooth-muscle-actin in co-cultivated HUASMCs upon high laminar FSS (3 N/m^2^) (magnification: 1∶400).

Scanning electron microscopy (SEM) was performed after stimulating the “*artificial artery*” for 24 h with low laminar FSS (0.1 N/m^2^). SEM pictures of the inside of a hollow fiber prove that endothelial cells are arranged as homogeneous cell cluster in a “hill and valley” morphology, whereby each cell can be recognized by the oval shaped nucleus characteristically placed in the center [50], [51] (**Figure 3A**). SEM pictures also unveil the typical spindle shaped morphology of smooth muscle cells growing on the outside of hollow fibers (**Figure 3B**). Cadherin-5 encodes for a cell adhesion molecule required for the organization of intercellular junctions exclusively and constitutively expressed in endothelial cells [52]. It is known to play an essential role in determining the endothelial cell morphology [53]. Confocal microscopic immunolocalization of Cadherin-5 demonstrates that co-cultivated HUVECs exposed to low laminar FSS (0.1 N/m^2^) for five days show the characteristic cobblestone like morphology (**Figure 3C**) [54]. Stimulating co-cultivated HUVECs with high laminar FSS (3 N/m^2^) for five days revealed an elongated morphology parallel to the direction of flow [55] upon Cadherin-5 staining (**Figure 3D**). Immunofluorescence staining of α-smooth-muscle-actin in HUASMCs co-colonized with low (**Figure 3E**) and high (**Figure 3F**) laminar shear stress stimulated HUVECs over a period of five days confirmed the characteristic smooth muscle cell morphology.

**Figure 4:**
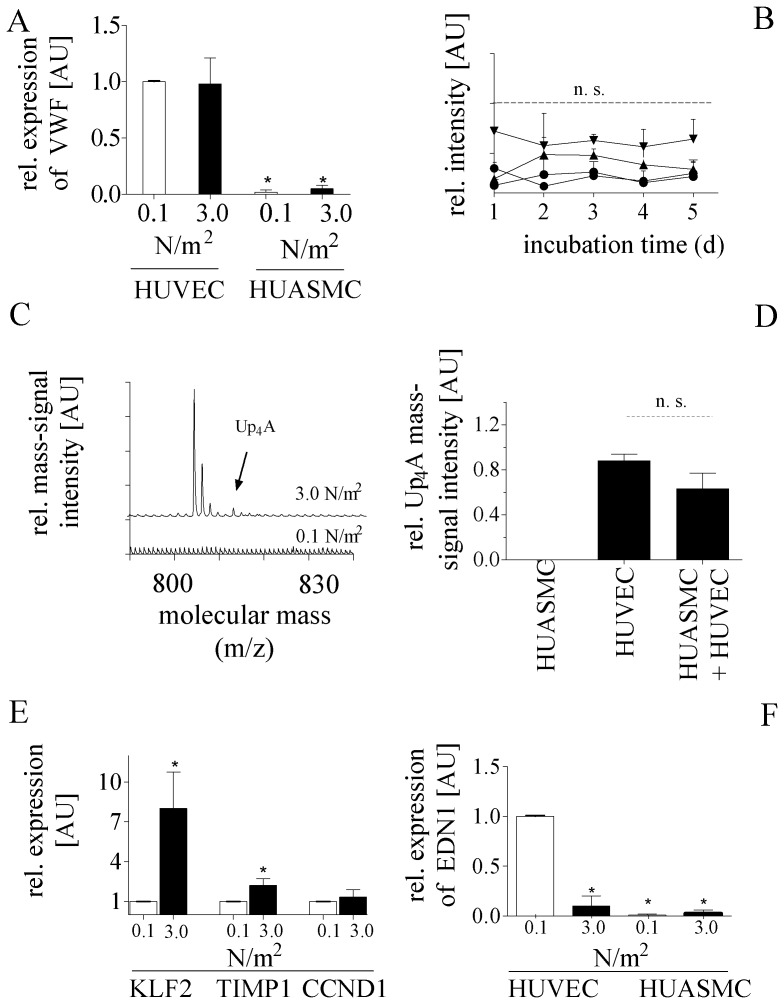
Validation of the “*artificial artery*” as *in vitro* co-culture system with arterial functional characteristics mimicking *in vivo* conditions of the vasculature. Semi-quantitative RT-PCR analysis showing the total VWF mRNA expression in co-colonized HUVECs and HUASMCs. RNA of both cell types was isolated separately. The expression of VWF in 0.1 N/m^2^ stimulated HUVECs was taken as reference. (**A**) MALDI mass spectrum from supernatants of the “*artificial artery”* after each day. Randomly selected peptides show constant molecular mass-signal intensities over a period of five days (abscissa: relative molecular mass m/z, z = 1; ordinate: relative intensity, arbitrary units). (**B**) MALDI mass spectrum of the supernatant of endothelial cells after stimulation with 3.0 N/m^2^ (upper spectrum) and without stimulation (0.1 N/m^2^) (lower spectrum) (abscissa: relative molecular mass, m/z, z = 1; ordinate: relative intensity, arbitrary units). (**C**) Relative mass-signal intensities of Up_4_A in secretomes isolated from HUVECs, HUASMCs, and HUVEC/HUASMC co-cultures in the “*artificial artery*” after stimulating with 3 N/m^2^ for five days. (**D**) Semi-quantitative RT-PCR analyses showing the total mRNA expression of KLF2, TIMP1, and CCND1 in co-colonized HUVECs exposed to 3 N/m^2^ for five days. The expression of each gene in 0.1 N/m^2^ stimulated HUVECs was taken as reference. (**E**) Semi-quantitative RT-PCR analysis showing the total EDN1 mRNA expression in co-colonized HUVECs (0.1 N/m^2^ vs. 3 N/m^2^) and HUASMCs (0.1 N/m^2^ vs. 3 N/m^2^). The expression of END1 in 0.1 N/m^2^ stimulated HUVECs was taken as reference.

The mRNA expression level of Van Willebrand factor (VWF) in co-cultured HUVECs stimulated with low (0.1 N/m^2^) vs. high (3 N/m^2^) laminar FSS for five days was not significantly different (**Figure 4A**). Comparing mRNA expression levels of VWF in co-cultivated HUVECs and HUASMCs demonstrated a negligible VWF expression in HUASMCs (**Figure 4A**).

To prove viability of our model, we analysed the stability of the secretion rate of randomly chosen peptides over a cultivation period of five days. We daily isolated co-culture secretomes and quantified the relative intensity of four randomly chosen molecular mass-signals by mass spectrometry as previously described [40], [56]. Since we detected no significant decrease or increase of those mass-signal intensities within a cultivation period of five days (**Figure 4B**), a constant secretion of the randomly chosen peptides can be concluded. These results demonstrated the strong viability of the model within a time-period of five days.

To investigate Up_4_A secretion upon high levels of laminar flow, the secretome of HUVEC mono-cultures stimulated with low (0.1 N/m^2^) and high (3 N/m^2^) levels of laminar FSS over a period of five days was isolated and further analysed by mass spectrometry [57]. Mass spectra of HUVEC mono-cultures stimulated with high laminar FSS (3 N/m^2^) for five days showed the intensity of the Up_4_A mass-signal at 814 Da (**Figure 4C**; upper mass spectrum). In the secretome of HUVEC mono-cultures stimulated with low laminar FSS (0.1 N/m^2^), no Up_4_A mass-signal could be detected (**Figure 4C** **;** lower mass spectrum).

To validate that Up_4_A is exclusively and constitutively secreted by vascular endothelial cells, the secretomes of FSS stimulated mono-cultured HUVECs, HUASMCs or co-cultured modules were isolated. **Figure 4D** shows the relative mass-signal intensities of Up_4_A in secretomes isolated from those modules after high laminar FSS stimulation. Up_4_A was detected in both HUVEC mono-culture and co-culture secretomes, but not in the secretome of HUASMC mono-cultures.

Investigating mRNA expression of mechano-regulated genes in HUVECs isolated from co-culture modules after high laminar FSS stimulation for five days, we could show a significant increase of Kruppel-like factor 2 (KLF2) mRNA expression in comparison to 0.1 N/m^2^ (**Figure 4E**). We were also able to show a significant induction of tissue inhibitor of matrix metalloprotease-1 (TIMP1) mRNA expression (**Figure 4E**). However, cyclin D1 (CCND1) mRNA expression, as marker for cell proliferation, did not change upon high laminar FSS stimulation (**Figure 4E**). This shows that co-cultured endothelial cells within the “*artificial artery*” are vital, mechanically responsive, and do not proliferate due to a quiescent phenotype.

EDN1 mRNA expression in co-cultured HUVECs exposed to high laminar FSS (3 N/m^2^) was significantly down-regulated after five days. This verifies that physiological levels of high laminar FSS applied to co-cultivated HUVECs are recognized as arterio-protective, homeostatic conditions. Comparing EDN1 mRNA expression levels in HUVECs and HUASMCs additionally confirmed that RNA of both cell types could separately be isolated without cross-contamination (**Figure 4F**). Affirming this, EDN1 mRNA expression in HUASMC mono-cultures was barely detectable (data not shown).

## Discussion

While Takei *et al.* [4], Ballermann *et al*. [58], and Ucuzian *et al.* [12] used endothelial cell mono-culture approaches for mimicking the *in vivo* situation of small arteries, co-culturing of endothelial cells and vascular smooth muscle cells as in the bioreactor “*artificial artery*” ensures a direct interaction of relevant cell types of the vascular system. Developing a co-culture module as presented in this study is a very complex process. Co-culture conditions have to be defined dependent on the hollow fiber structure and a sufficient supply of both cell types with nutrients and oxygenation. The interaction of different cell types is furthermore essential to investigate physiological and pathophysiological effects, since local concentrations of mediators secreted by the cells may be significantly higher in case of a direct interaction than in the resulting plasma concentrations.

Some years ago, Redmond *et al.* [59] have described an *in vitro* co-culture model similar to the “*artificial artery*”. Comparing both models, our cell seeding procedure is more efficient, less complicated, and confluence of both cell types is already achieved after one day. The model described by Redmond *et al.* [59] additionally lacked of its functional validation regarding biomechanical response, physiological and pathophysiological aspects as the secretion of endothelial mediators, immunofluorescence staining of proteins specifically expressed in endothelial or smooth muscle cells, staining of cell nuclei, and mRNA expression analysis. We have used the aforementioned methods as well as SEM and MALDI TOF/TOF mass spectrometry to verify the potentiality of our *in vitro* co-culture module as amplificator of cell biomarkers amount whereby we always compared results upon the application of two different physiological relevant shear stress levels (0.1 N/m^2^ and 3 N/m^2^).

Hoechst 33342 staining and SEM of HUVECs and HUASMCs of co-culture modules revealed that confluence and the typical morphology of both cell types [50], [51] have already been reached after 24 h. Immunocytochemistry analyses of Cadherin-5 as marker expressed in HUVECs and α-smooth-muscle-actin as HUASMC marker demonstrated a proper morphology of both cell types after a cultivation period of five days. Cadherin-5 is a calcium-dependent cell–cell adhesion glycoprotein regulating the cohesion and organization of intercellular junctions. It mostly interacts with another Cadherin-5 protein in a homophilic manner [60]. The typical “zipper like” structure of Cadherin-5 homodimers [61] upon low laminar FSS (0.1 N/m^2^) could be shown by immunocytochemistry. The application of high laminar FSS (3 N/m^2^) resulted in a highly elongated cell shape and orientation or alignment of HUVECs parallel to the direction of flow. We could furthermore show that the expression of α-smooth-muscle-actin in co-cultivated HUASMCs is independent of the applied FSS.

Analysing mono-and co-culture secretomes by MALDI TOF/TOF mass spectrometry verified viability of our model within an experimental range of up to five days and furthermore confirmed the physiological activity of HUVECs as a function of the applied shear stress through analysing Up_4_A secretion rate.

VWF is known to be constitutively produced in the endothelium. It functions as antihemophilic factor carrier and platelet-vessel wall mediator in the blood coagulation system [62]. Galbusera *et al.* found that the VWF release of HUVECs upon high laminar FSS increased without an up-regulation of VWF mRNA expression [63]. We could confirm theses data and furthermore showed that the isolation of HUVEC and HUASMC RNA was performed without cross-contamination.

To validate shear stress responsiveness of our co-culture system, we analysed total mRNA expression of the mechano-sensitive genes KLF2, EDN1, TIMP1, and CCND1, as marker for cell proliferation, in HUVECs upon high laminar FSS (3 N/m^2^) relative to 0.1 N/m^2^.

KLF2, a zinc finger protein acting as central transcriptional regulator, is known as key shear stress-induced transcription factor activating anti-inflammatory and anti-coagulant proteins. It also regulates pro-inflammatory and anti-fibrinolytic genes through the inhibition of pro-inflammatory transcription factors [64]. KLF2 contains a shear stress responsive element in its promoter region whereby binding of transcription factors such as PCAF (P-300/cAMP-response element-binding protein-binding protein-associated factor) and heterogeneous nuclear ribonucleoprotein D, components of the shear stress regulatory complex, leads to the up-regulation of KLF2 expression *in vivo* and *in vitro* upon physiological levels of laminar FSS [65], [66]. Confirming this, we were able to show a significant up-regulation in the expression of KLF2 upon high laminar FSS applied for five days. Dekker *et al.* have shown that KLF2 over-expression induces eNOS expression and also confirmed decreased EDN1 expression regulated through KLF2 [67]. KLF2 gene expression is therefore suggested to be elementary for the prevention of CVDs [65].

Over-expression of EDN1, a potent vasoconstrictor peptide derived by the endothelium, has been implied in the pathogenesis of artherosclerosis and hypertension [68]. Malek *et al.* could show that EDN1 mRNA expression is down-regulated upon high laminar FSS [69]. We could support these findings and showed a down-regulation of EDN1 mRNA expression after the application of high laminar FSS revealing its role in homeostasis.

TIMP1 is transcriptionally up-regulated in a model for coronary arteriogenesis *in vivo* [70]. It has been discussed as mediator for proliferation and protection from apoptosis and furthermore described as marker for early-phase arteriogenesis in the rat brain [71]. Confirming this, we found that TIMP1 mRNA expression was up-regulated upon high laminar FSS.

CCND1 is required for cell cycle G1/S transition and was therefore used to investigate proliferation of endothelial cells. Earlier findings show that CCND1 mRNA and protein levels are unaffected by physiological levels of shear stress [72], [73]. We were able to confirm that CCND1 mRNA expression of endothelial cells did not change upon shear stress indicating a quiescent endothelial phenotype.

Taken together, these results demonstrate that we have established an *in vitro* co-culture system with arterial functional characteristics which mimics *in vivo* conditions of the vasculature. To research processes of early-phase arteriogenesis, we stimulated the cells up to five days with two physiological levels of laminar FSS whereby the cells were still viable and showed biomechanical response. It would also be feasible to investigate long-term effects (e.g. remodelling processes) using an incubation period longer than five days or to apply pulsatile FSS using a different pump system. In conclusion, the “*artificial artery*” is a reliable *in vitro* model to investigate cell-cell specific interactions, molecular mechanisms, and time-dependent effects under physiological flow conditions. It helps to reduce the number of animal experiments investigating CVDs according to the 3R concept of Russel and Burch [74].

The bioreactor “*artificial artery*” combined with all functional assays established in this study as well as the end-point analyses of known biomarkers enables a high spectrum of possible applications e.g. to screen the role of known and unknown pharmacological substances and pro-arteriogenic factors in CVDs and to analyse the biochemistry behind. Underlying signalling pathways can furthermore be investigated to provide insights into molecular mechanisms of CVDs. To improve the diagnosis and/or even treatment of CVDs, yet unknown biomarkers and mediators can be isolated from the corresponding secretomes. The secretome of the “*artificial artery*” for instance could be screened for (patho-) physiological activities utilizing additional *in vitro* assays, such as assays to investigate the generation of reactive oxygen species, vascular calcification etc. It is also possible to combine the “*artificial artery*” with other preceding *in vitro* systems (e.g. metabolom reactors) to analyse molecular mechanisms of multiple diseases such as endothelial dysfunction, diabetes mellitus, renal failure or heart failure.

In conclusion, the “*artificial artery”* is applicable for the analysis of vascular biomarkers and mediators involved in proliferation and/or remodelling processes, the release of soluble peptides via mass spectrometric analysis (secretome analysis), the identification of mediators involved in these processes, and RNA expression analyses of genes regulated by shear stress.

## Supporting Information

Figure S1 **Optimization of HUVEC/HUASMC co-culture conditions.** (**A**) Hoechst 33342 staining of HUVEC nuclei colonized onto polyethersulfone and polypropylene flat sheet membranes. Corning membrane inserts were used as positive control (magnification: 1∶200). (**B**) Representative MALDI mass spectrum of the supernatant of HUVECs colonized onto uncoated flat sheet membranes (abscissa: relative molecular mass, m/z, z = 1; ordinate: relative mass-signal intensity, arbitrary units). (**C**) Representative MALDI mass spectrum of the HUVEC supernatant from fibronectin-coated flat sheet membranes (abscissa: relative molecular mass, m/z, z = 1; ordinate: relative mass-signal intensity, arbitrary units). Figure S1B and S1C are equally scaled. (**D**) Confocal microscopic immunolocalization of ß-Catenin in HUVECs colonized onto fibronectin-coated polypropylene flat sheet membranes which were pre-stimulated with different wash protocols. Hoechst dye solution was used for fluorescent staining of nuclei (blue) (magnification: 1∶630).
(DOC)

Table S1 Membrane characteristics of flat sheet membranes.
(DOC)
