# Supplementary figures and images for: The “Artificial Artery” as In Vitro Perfusion Model
Source: PLoS One. 2013 Mar 7;8(3):e57227. doi: 10.1371/journal.pone.0057227 (PMC3591414; doi:10.1371/journal.pone.0057227)

**Figure S1:**

**
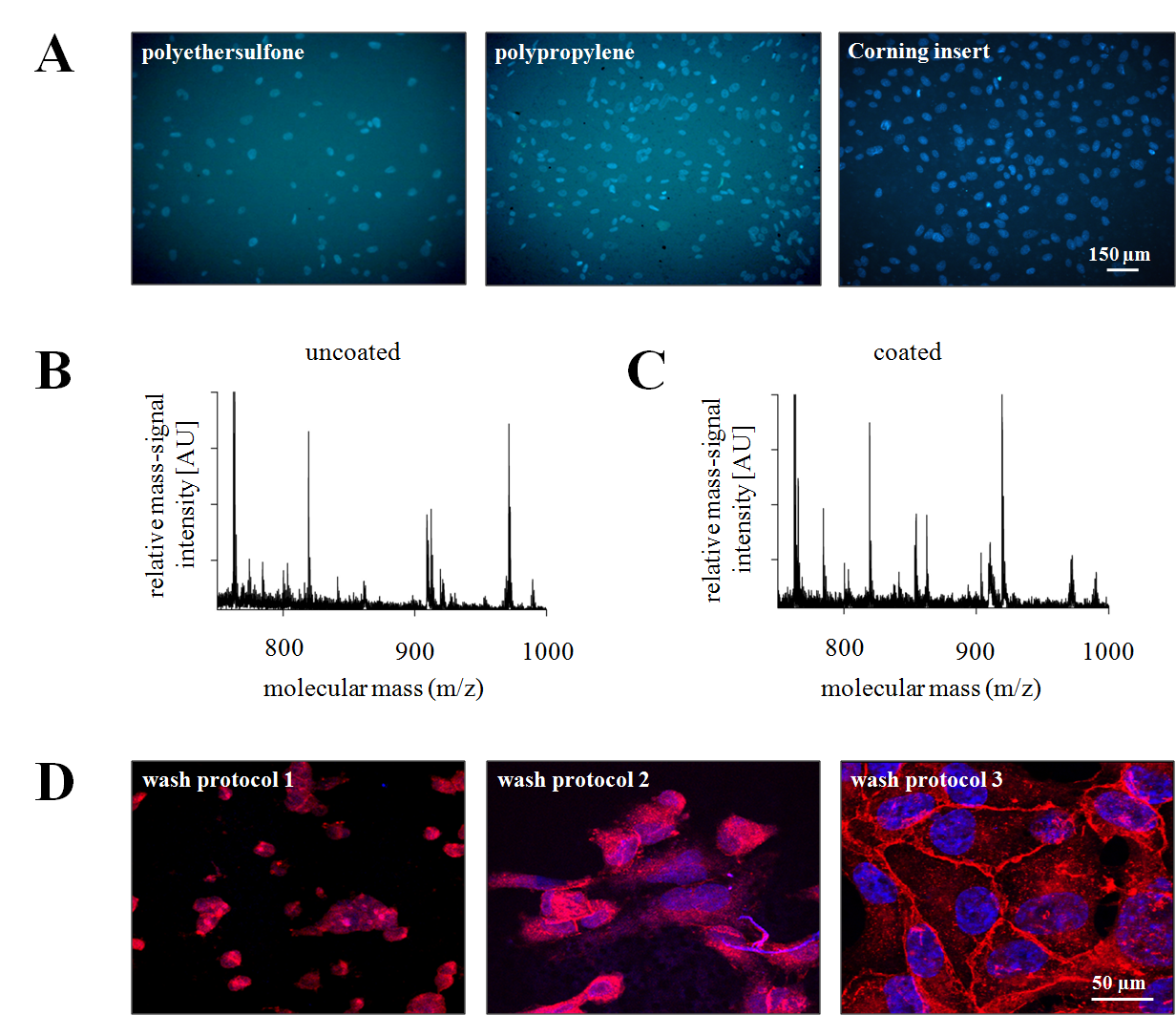
**

Supplement: Figure S1 — Optimization of HUVEC/HUASMC co-culture conditions. (A) Hoechst 33342 staining of HUVEC nuclei colonized onto polyethersulfone and polypropylene flat sheet membranes. Corning membrane inserts were used as positive control (magnification: 1∶200). (B) Representative MALDI mass spectrum of the supernatant of HUVECs colonized onto uncoated flat sheet membranes (abscissa: relative molecular mass, m/z, z = 1; ordinate: relative mass-signal intensity, arbitrary units). (C) Representative MALDI mass spectrum of the HUVEC supernatant from fibronectin-coated flat sheet membranes (abscissa: relative molecular mass, m/z, z = 1; ordinate: relative mass-signal intensity, arbitrary units). Figure S1B and S1C are equally scaled. (D) Confocal microscopic immunolocalization of ß-Catenin in HUVECs colonized onto fibronectin-coated polypropylene flat sheet membranes which were pre-stimulated with different wash protocols. Hoechst dye solution was used for fluorescent staining of nuclei (blue) (magnification: 1∶630). (DOC) [file pone.0057227.s001.doc]
