# Supplementary material for: The “Artificial Artery” as In Vitro Perfusion Model
Source: PLoS One. 2013 Mar 7;8(3):e57227. doi: 10.1371/journal.pone.0057227 (PMC3591414; doi:10.1371/journal.pone.0057227)
Supplement: Table S1 — Membrane characteristics of flat sheet membranes. (DOC) [file pone.0057227.s002.doc]

**Table S1:** Membrane characteristics of flat sheet membranes

|  | **Wall thickness**  **[µm]** | **Maximum pore size [µm]** |
| --- | --- | --- |
| polypropylene | 150 | 0.47 |
| polyethersulfone | 100 | 0.5 |
| polyethylene | 110 | 0.26 |
| polyetherester | 19 | 8 |
| polyethylene terephthalate | 15 | non-porous |
| polytetrafluoroethylene | N/A | 0.2 |
| polyamide | 140 | 0.1 |
